# Accuracy Evaluation of The Depth of Six Kinds of Sperm Counting Chambers for both Manual and Computer-Aided Semen Analyses

**DOI:** 10.22074/ijfs.2015.4612

**Published:** 2015-12-23

**Authors:** Jin-Chun Lu, Ru-Qian Yue, Rui-Xiang Feng, Ling-Zhu Kong, Yuan-Cheng Xu

**Affiliations:** 1 Department of Laboratory Medicine, Nanjing Hospital, Jiangsu Corps, the Armed Police Force, PLA, Nanjing 210028, Jiangsu, China; 2 Geoffrey Laboratory for Semen Analysis, Jiangsu Jingcheng Pharmaceuticals Co., Ltd., Nanjing 210036, Jiangsu, China

**Keywords:** Depth, Measurement, Sperm Counting Chamber

## Abstract

**Background:**

Although the depth of the counting chamber is an important factor influencing sperm counting, no research has yet been reported on the measurement and comparison of the depth of the chamber. We measured the exact depths of six kinds of sperm counting chambers and evaluated their accuracy.

**Materials and Methods:**

In this prospective study, the depths of six kinds of sperm counting chambers for both manual and computer-aided semen analyses, including Makler (n=24), Macro (n=32), Geoffrey (n=34), GoldCyto (n=20), Leja (n=20) and Cell-VU (n=20), were measured with the Filmetrics F20 Spectral Reflectance Thin-Film Measurement System, then the mean depth, the range and the coefficient of variation (CV) of each chamber, and the mean depth, relative deviation and acceptability of each kind of chamber were calculated by the closeness to the nominal value. Among the 24 Makler chambers, 5 were new and 19 were used, and the other five kinds were all new chambers.

**Results:**

The depths (mean ± SD, μm) of Makler (new), Macro and Geoffrey chambers were 11.07 ± 0.41, 10.19 ± 0.48 and 10.00 ± 0.28, respectively, while those of GoldCyto, Leja and Cell-VU chambers were 23.76 ± 2.15, 20.49 ± 0.22 and 24.22 ± 2.58, respectively. The acceptability of Geoffrey chambers was the highest (94.12%), followed by Macro (65.63%), Leja (35%) and Makler (20%), while that of the other two kinds and the used Makler chamber was zero.

**Conclusion:**

There existed some difference between the actual depth and the corresponding nominal value for sperm counting chambers, and the overall acceptability was very low. Moreover, the abrasion caused by the long use, as of Makler chamber, for example, may result in unacceptability of the chamber. In order to ensure the accuracy and repeatability of sperm concentration results, the depth of the sperm counting chamber must be checked regularly.

## Introduction

Semen analysis counts significantly among laboratory examinations in andrology, and sperm concentration is one of the basic parameters of routine semen analysis. Although the world health organization (WHO) laboratory manual for the examination and processing of human semen recommends the Neubauer haemocytometer chamber as a sperm counting chamber, and provides methods for quality control of sperm counting (1), many other sperm counting chambers, including DROP, Standard Count, Cell Vision, MicroCell, 2X-CEL, Makler, JCD, Burker, CellVU, Leja, Macro, GoldCyto and Geoffrey, a newly designed chamber from the Geoffrey laboratory, have also been introduced into andrology laboratories. The precision and accuracy of these sperm counting chambers have been widely evaluated and compared (2,13), and most researches have demonstrated wide differences in the results of counting between different sperm counting chambers. 

Many factors contribute to the differences in the results of sperm counting, such as sample mixing, loading, environmental temperature, etc. (14,16). However, the depth of the counting chamber is an important factor influencing sperm counting and motility (17,18). A significant positive correlation was found between the depth of the sperm chamber and bead concentration (r=0.997, P<0.01) as well as between an unacceptable sperm chamber and incorrect result of sperm concentration, and the error was directly proportional to that of the sperm chamber depth (17). If the depth of the sperm counting chamber used for semen analysis does not meet the requirements of permitted error, the accuracy of the results of sperm concentration for all semen samples in the laboratory will be inevitably affected, which is intolerable for both patients and clinicians. Even if a brand is chosen as the standard counting chamber, different batches of products by the same manufacturer might differ significantly in their depths (19). Moreover, the existing researches have raised little doubt about and paid little attention to the accuracy of the chamber’s depth. Extensive literature retrieval has failed to identify any reported studies on the measurement and comparison of the depth of sperm counting chambers. Therefore, we measured the exact depths of six kinds of sperm counting chambers which are widely used for both manual and computer-aided semen analyses (12), and evaluated their accuracy. The detailed report is as follows. 

## Materials and Methods

### Materials

The Filmetrics F20 Spectral Reflectance Thin-Film Measurement System was provided by the DYMEK Company, USA, which has a measurable range from 15 nm to 70 μm and the lowest detectable limit of 1 nm. The system can be used to measure the thickness of the thin-film by analyzing the reflected light off its two surfaces and then calculate the spectral reflectance at a range of wavelength. A reflectance calculator gives the thickness value of the thin-film based on the complex-matrix form of the Fresnel equations. A film of standard thickness (serial number: National Institute of Standards and Technology (NIST)-12016), SiO_2_ on Si Standard Thickness=(725.9 ± 1.1) Å, was certified by the NIST, USA. Five 10 μm-deep Makler sperm chambers were purchased from Sefi Medical Instruments, Israel. Thirty-two Macro sperm counting chambers and 34 Geoffrey chambers, both of 10 μm depth, were provided by Jiangsu Rich Life Science Instrument Co., Ltd (Xuzhou, China). Twenty Gold- Cyto chambers (Microptic s.l. 321 6˚ 4^a^-08029, Barcelona, Spain) and 20 Leja chambers (Leja Products B.V. Luzemestraat 10 2153 GN, Nieuw Vennep, The Netherlands), both 20 μm in depth, were bought from Guangdong Youning Trade Co., Ltd (Guangzhou, China). Twenty Cell-VU chambers of 20 μm in depth (Millennium Sciences Inc., USA) were purchased from Nanjing Yu’an Instrument Co., Ltd (Nanjing, China). In addition, nineteen 10 μm-deep Makler sperm counting chambers (Sefi Medical Instruments, Israel), which had been used for several months or years, were obtained from different andrology laboratories in China. Makler, Macro and Geoffrey chambers are manufactured with the base glass and separate cover glass and can be used repeatedly. GoldCyto and Leja chambers belong to the disposable kind with fixed depth. Cell-VU chamber, also made from the base glass and separate cover glass, can be used repeatedly or as a disposable chamber.

## Methods

This was a prospective study. First, the Filmetrics F20 Spectral Reflectance Thin-Film Measurement System was calibrated with the standard film according to the operating instruction. Then, six kinds of sperm counting chamber, including Makler, Cell-VU, Leja, Macro, GoldCyto and Geoffrey, were numbered randomly, cleansed with a solution (ether: alcohol, 7:3), and confirmed to be devoid of impurity under a microscope. Finally, the depths of the upper, lower, left, right and central parts of each chamber were measured, and the average depth, SD and the range of depth (maximum value minus minimum value) of each chamber was calculated automatically, all with the Filmetrics F20 System according to the operating instructions. All the sperm counting chambers were measured by one technician, and the supervision and verification of the results were conducted by another. 

## Statistical analysis

All the data obtained were put into an Excel table for calculation of the mean depth, the range of depth, and coefficient of variation (CV) of each chamber. For those with double chambers, the paired t test was used to compare the difference between the two; for those with more than two chambers, the comparison was made by the LSD-t test, and the new and used Makler chambers were compared by the independent-sample t test. The SPSS (SPSS Inc., USA) version 11.0 software was used for analyses, and statistically significant difference was designed to be P<0.05. For a 5% permitted error, the chambers with a measured depth of 10 ± 0.5 μm or 20 ± 1 μm were judged as compatible with a nominal value of 10 μm or 20 μm, respectively, followed by assessment of the acceptability of different sperm counting chambers. 

## Results

The depths of all the six kinds of sperm counting chambers were measured. The results are shown in table 1. 

The average depth of the 32 Macro sperm counting chambers (with a single chamber and nominal depth of 10 μm) was 10.19 μm, and CV was 4.71%. The range of the mean and CV of the five depth measurements of Macro chambers were 0.14 μm and 0.58%, respectively, and 65.63% of the 32 chambers were within acceptable limits. 

The Makler sperm counting chamber is also a single chamber unit with depth of 10 μm. The mean depth of the five new Makler chambers was 11.07 μm, with a relative deviation of 10.7%, exceeding the allowable range of 5%, and that of the 19 used Makler chambers was 12.72 μm, with a relative deviation of 27.2%, far exceeding the allowable range. Moreover, the CV (8.49%) for all used chambers and the mean range (0.43 μm) and CV (1.33%) of the depth between the 5 points were obviously higher than those of the Macro chambers. The acceptability was 20% for the new Makler chambers, and zero for the used ones. 

A Cell-VU sperm counting chamber includes two 20 μm-deep chambers. The mean depth and relative deviation of the 20 left chambers were 23.94 μm and 19.7%, and those of the 20 right ones were 24.49 μm and 22.45%, respectively. Although there was no significant difference in depth between the left and right chambers (t=-1.231, P=0.233), the CVs (12.36 vs. 10.41%) for all the chambers on either side and the mean ranges (1.64 μm vs. 2.21 μm) and CVs (2.66 vs. 3.47%) of the depth between the 5 points in each chamber were very high. The acceptability was 5% (1/20) for both the left and right chambers. If it was required that the errors of the two chambers in one set to be within the allowable range, the acceptability of the Cell-VU chamber was zero.

A Geoffrey sperm counting chamber contains two 10 μm-deep chambers. The mean depth of the 34 left chambers was 10.01 μm and that of the 34 right ones was 9.99 μm, with no significant difference between them (t=0.801, P=0.429). The CVs (2.90 vs. 2.80%) for all the chambers on either side, and the mean ranges (0.11 vs. 0.11 μm) and CVs (0.45 vs. 0.47%) of the depth between the 5 points in each chamber were all very low. The acceptability values of the left and right chambers were 100 and 94.12%, respectively. If it was required that the errors of the two chambers in one set to be within the allowable range, the acceptability of the Geoffrey chamber was 94.12%. 

A GoldCyto sperm counting chamber contains four 20 μm-deep chambers (A-D). The mean depths of chambers A, B, C and D of the 20 GlodCyto sets were 22.19, 24.79, 24.96 and 23.11 μm, respectively. The corresponding relative deviations were 10.95, 23.95, 24.8 and 15.55%, respectively, with a total deviation of 18.8%, all far exceeding the allowable range of 5%. However, the depths of chambers B and C were significantly greater than those of A and D (P<0.05), although there was no significant differences in depth either between B and C or between A and D. The acceptability values of chambers A, B, C, and D were 30, 5, 5 and 10%, respectively. If one of the chambers was unacceptable, the whole unit was appraised as unacceptable and the acceptability of the GoldCyto sperm counting chamber was zero. 

A Leja sperm counting chamber consists of eight 20 μm-deep chambers (A-H). The mean depths of chambers A, B, C, D, E, F, G, and H of the 20 Leja chambers were 20.99, 20.37, 20.30, 20.34, 20.88, 20.31, 20.17 and 20.55 μm, respectively. The corresponding relative deviations were 4.95, 1.85, 1.5, 1.7, 4.4, 1.55, 0.85 and 2.75%, respectively, indicating that all were within the allowable range of 5%. However, the depths of chambers A and E were significantly greater than those of B, C, D, F, G, and H (P<0.05), and the depth of H was greater than that of G (P<0.05). The acceptability values of chambers A, B, C, D, E, F, G, and H were 65, 95, 95, 100, 65, 100, 95 and 95%, respectively. However, if one of the chambers was unacceptable, the whole unit was appraised as unacceptable and the acceptability of the Leja sperm counting chamber was 35%. 

**Table 1:** Determination results of six kinds of sperm counting chambers’ depth


Sperm counting chamber	n	Depth of chamber(range) (μm)	CV between chambers (%)	Variation of depth between 5 points (Max-Min, μm)	CV between five points (%)	Acceptability(%)

Macro	32	10.19 ± 0.48	4.71	0.14	0.58	65.63
		(9.25-10.95)		(0.01-0.37)	(0.06-2.55)	
Makler (New)	5	11.07 ± 0.41	3.70	0.086	0.30	20
		(10.50-11.60)		(0.04-0.14)	(0.15-0.60)	
Makler (Be used)	19	12.72 ± 1.08	8.49	0.43	1.33	0
		(11.32-14.64)		(0.03-1.27)	(0.09-3.88)	
Cell-VU	20	24.22 ± 2.58	10.65			0
		(18.21-26.83)				
Left	20	23.94 ± 2.96	12.36	1.64	2.66	5
		(17.01-28.34)		(0.35-3.12)	(0.66-4.67)	
Right	20	24.49 ± 2.55	10.41	2.21	3.47	5
		(18.68-27.83)		(0.58-4.09)	(0.84-6.33)	
Geoffrey	34	10.00 ± 0.28	2.80			94.12
		(9.54-10.62)				
Left	34	10.01 ± 0.29	2.90	0.11	0.45	100
		(9.54-10.62)		(0.02-0.21)	(0.11-0.85)	
Right	34	9.99 ± 0.28	2.80	0.11	0.47	94.12
		(9.56-10.57)		(0.01-0.36)	(0.05-1.61)	
GoldCyto	20	23.76 ± 2.15	9.06			0
		(18.43-26.63)				
A	20	22.19 ± 2.62	11.81	1.81	3.27	30
		(17.68-27.68)		(0.39-4.13)	(0.88-5.59)	
B	20	24.79 ± 2.52	10.17	1.99	3.23	5
		(19.30-29.31)*		(0.24-4.80)	(0.43-7.52)	
C	20	24.96 ± 3.10	12.42	1.79	2.96	5
		(18.79-29.67)*		(0.37-3.90)	(0.69-5.96)	
D	20	23.11 ± 2.89	12.51	1.93	3.22	10
		(17.97-27.81)		(0.15-4.29)	(0.24-6.67)	
Leja		20.49 ± 0.22	1.06			35
		(20.27-21.34)				
A	20	20.99 ± 0.39	1.86	0.45	0.85	65
		(20.53-21.71)		(0.13-0.80)	(0.25-1.67)	
B	20	20.37 ± 0.58	2.85	0.17	0.33	95
		(19.99-22.68)#		(0.05-0.42)	(0.10-0.85)	
C	20	20.30 ± 0.34	1.67	0.15	0.29	95
		(19.99-21.67)#		(0.03-0.44)	(0.05-0.87)	
D	20	20.34 ± 0.14	0.69	0.18	0.35	100
		(20.15-20.65)#		(0.04-0.42)	(0.07-0.96)	
E	20	20.88 ± 0.42	2.01	0.31	0.59	65
		(20.28-21.64)		(0.11-0.60)	(0.21-1.29)	
F	20	20.31 ± 0.26	1.28	0.21	0.44	100
		(20.00-20.92)#		(0.07-0.38)	(0.14-0.86)	
G	20	20.17 ± 0.46	2.28	0.11	0.22	95
		(19.89-22.07)#		(0.03-0.4)	(0.04-0.69)	
H	20	20.55 ± 0.30	1.46	0.33	0.63	95
		(20.23-21.49)#∆		(0.04-0.61)	(0.08-1.14)	


For Cell-VU and Geoffrey chambers, the difference between the left and right chambers was compared with paired t test. There was no significant difference in depth between the left and right chambers of Cell-VU chambers (t=-1.231, P=0.233) and between the left and right chambers of Geoffrey chambers (t=0.801, P=0.429). The comparison of new and used Makler chambers was analyzed with independent-samples t test, and there was significant difference between them (t=5.325, P<0.001). For GoldCyto and Leja chambers, the difference between the chambers was compared with LSD-t test. CV; Coefficient of variation *; P<0.05 vs. A and D in the same group, #; P<0.05 vs. A and E in the same group and Δ; P<0.05 vs. G in the same group. There was no significant difference between other chambers in the same group (P>0.05).

### Discussion

The WHO Manual (5 ^th^edition) emphasizes regular measurement of the depth of the sperm counting chamber, but in practice, it is never measured since its purchase till worn out. Moreover, there is a lack of methods to identify the chambers and specific measures for the implementation of quality control for the chambers. 

Therefore, we measured the depth of six kinds of sperm counting chambers with the Filmetrics F20 Spectral Reflectance Thin-Film Measurement System, which has been widely used to measure the thickness of some thin film materials (20,22). 

The results of measurement of the six kinds of chambers showed that no chamber, either 10 μm or 20 μm deep, was 100% acceptable. The highest acceptability was 94.12%, as exhibited by the Geoffrey sperm counting chamber, a new type developed by Jiangsu Rich Life Science Instrument Co., Ltd, China, with an optical glass plate embedded in a metal frame base and a cover plate containing a metal frame inlaid with an optical coverslip. There are four ruby spherical pillars and two independent chambers on the optical glass plate. The relatively high acceptability of Geoffrey chambers may be attributed to the fine polishing processing and calibration of each plane of the optical glass plate, strict quality control measures for the adjustment of the chamber’s depth, and precise detection of the depth of each chamber before dispatched from the factory. 

The acceptability of the Macro chamber was 65.63%, and the average range between the 5 points was 0.14 μm, a little lower than that of the Geoffrey chambers. The Macro chamber, similar to the Makler chamber in design, except for its three ruby spherical pillars instead of four glass columns in the latter, has been widely used in andrology laboratories in China. Using the principle of three points defining a plane, the production of the Macro chamber may have dismissed Newton’s rings to ensure a closer contact of the cover plate with the three supporting points. 

The Leja chamber comprises eight 20 μm-deep chambers. Although its overall acceptability value was only 35%, the acceptability of each chamber was high. Therefore, in order to make all chambers meet the requirements of the allowable error, the standard of the production process must be relatively high. 

The acceptability of the new Makler chambers was 20%, while that of the other three kinds, including the used Makler, Cell-VU, and GoldCyto chambers, was zero, which may be attributable to the lack of strict control of chamber measurement or the use of an inaccurate measurement method at delivery inspection. In our study, the relative deviations of the left and right chambers of the Cell-VU unit were around 20%, and those of chambers B and C of the GoldCyto unit above 20%, indicating that all significantly higher than the allowable error range of 5%. The highest acceptability of all the chambers of the Makler, Cell-VU and GoldCy-to was only 30%. The abrasion from long use, as of the Makler chamber, may be one of the reasons for the unacceptable chambers. 

## Conclusion

There exists some difference between the actual depth and the corresponding nominal value of sperm counting chambers from different manufacturers, and the difference far exceeds the acceptable (95% CI) range for most of the chambers, which inevitably results in a large variation in sperm concentration in clinical application. Therefore, in order to ensure the accuracy and repeatability of semen analysis results, the depth of the sperm counting chamber must be checked regularly, even though strictly measured at delivery inspection, and unqualified chambers must be rejected. In addition, the measurement report for each sperm counting chamber must be attached to the product for an andrology laboratory. 
